# Attitudes toward Placebo-Controlled Clinical Trials of Patients with Schizophrenia in Japan

**DOI:** 10.1371/journal.pone.0143356

**Published:** 2015-11-24

**Authors:** Norio Sugawara, Masamichi Ishioka, Shoko Tsuchimine, Koji Tsuruga, Yasushi Sato, Hanako Furukori, Shuhei Kudo, Tetsu Tomita, Taku Nakagami, Norio Yasui-Furukori

**Affiliations:** 1 Aomori Prefectural Center for Mental Health and Welfare, Aomori, Japan; 2 Department of Neuropsychiatry, Hirosaki University School of Medicine, Hirosaki, Japan; 3 Department of Psychiatry, Hirosaki-Aiseikai Hospital, Hirosaki, Japan; 4 Department of Psychiatry, Seihoku-Chuoh Hospital, Goshogawara, Japan; 5 Department of Psychiatry, Kuroishi-Akebono Hospital, Kuroishi, Japan; 6 Department of Neuropsychiatry, Odate Municipal General Hospital, Odate, Japan; Maastricht University, NETHERLANDS

## Abstract

**Background:**

Although the use of placebo in clinical trials of schizophrenia patients is controversial because of medical and ethical concerns, placebo-controlled clinical trials are commonly used in the licensing of new drugs.

**Aims:**

The objective of this study was to assess the attitudes toward placebo-controlled clinical trials among patients with schizophrenia in Japan.

**Method:**

Using a cross-sectional design, we recruited patients (n = 251) aged 47.7±13.2 (mean±SD) with a DSM-IV diagnosis of schizophrenia or schizoaffective disorder who were admitted to six psychiatric hospitals from December 2013 to March 2014. We employed a 14-item questionnaire specifically developed to survey patients' attitudes toward placebo-controlled clinical trials.

**Results:**

The results indicated that 33% of the patients would be willing to participate in a placebo-controlled clinical trial. Expectations for improvement of disease, a guarantee of hospital treatment continuation, and encouragement by family or friends were associated with the willingness to participate in such trials, whereas a belief of additional time required for medical examinations was associated with non-participation.

**Conclusions:**

Fewer than half of the respondents stated that they would be willing to participate in placebo-controlled clinical trials. Therefore, interpreting the results from placebo-controlled clinical trials could be negatively affected by selection bias.

## Introduction

Schizophrenia is a severe mental illness that generally appears in late adolescence and affects approximately 1% of the population worldwide. Symptoms of schizophrenia are classified into three broad categories of positive symptoms (delusions, hallucinations, and disordered thoughts), negative symptoms (restricted affect and drive), and impairments in cognitive function [1, 2].

Current antipsychotic medications typically alleviate positive symptoms [3] and delay relapse [4] in patients with schizophrenia. However, negative symptoms [5] and impaired cognitive function [6] often persist despite currently available medications and frequently hinder patients’ return to full functioning. Furthermore, current antipsychotic medications, even second-generation antipsychotics, have significant side effects that may impair the health [7, 8] and quality of life [9] of schizophrenia patients. Because of the unsatisfactory outcomes following pharmacotherapy for schizophrenia, research is needed to compare the efficacy of new drugs in clinical trials.

In Japan, to obtain approval from the Pharmaceutical and Medical Devices Agency (PMDA), the agency requires significant proof of the efficacy of the drug based on adequate controlled trials. In the 2000s, the PMDA began to require placebo-controlled trials for new drug applications [10]. However, the use of placebo in clinical trials with schizophrenia patients is controversial because of medical and ethical concerns [11, 12]. Furthermore, the attitudes of patients with schizophrenia toward placebo-controlled clinical trials are a relatively unexplored yet critical factor.

The objectives of this investigation were (1) to obtain information about patients’ attitudes toward placebo-controlled clinical trials and (2) to assess factors related to the willingness to participate in placebo-controlled clinical trials among patients with schizophrenia in Japan. To the best of our knowledge, this article presents the first study conducted with an Asian population.

## Method

### Ethics Statement

The data collection for this study (2013–324) was approved by the Ethics Committee of the Hirosaki University School of Medicine, and all subjects provided written informed consent before participating in this study. The capacity of the patients to provide informed consent was assessed by their treating psychiatrists. Surrogate consent procedure was not employed for this study.

### Participants

This study was conducted between December 2013 and March 2014. The subjects included 251 outpatients (130 males and 121 females) at six psychiatric hospitals in Japan who were diagnosed with either schizophrenia or schizoaffective disorder based on the DSM-IV. The diagnoses of the patients were recorded based on their medical charts.

The demographic data (age, sex, duration of education) and medical history of the subjects were obtained from their medical records. Of the 251 patients in the study, 81 were receiving antipsychotic combination therapy, and 162 were receiving antipsychotic monotherapy. Medication-related information was lacking for 8 patients. Of the 162 patients receiving antipsychotic monotherapy, 146 were taking second-generation antipsychotics, and 16 patients were taking traditional antipsychotics.

The Clinical Global Impressions-Severity of Illness (CGI-S) Scale is a well-established research rating tool used to measure symptom severity that is applicable to all psychiatric disorders and can easily be used by practicing clinicians. The CGI-S uses ratings from 1 (normal, not at all ill) to 7 (among the most extremely ill patients). These ratings are based on all available information, including knowledge of the patient’s history, psychosocial circumstances, symptoms, and behavior, as well as the impact of the symptoms on the patient’s ability to function [13].

The Global Assessment of Functioning (GAF) is a scoring system (from 0 to 100) used by mental health clinicians to subjectively evaluate a patient's social, occupational, and psychological functions. The clinician assesses either the symptom severity or the level of functioning, depending on which is the most severe. Higher GAF scores indicate that participants either have less severe symptoms or have higher functioning [14].

After reviewing the relevant literature and extant guidelines [15, 16], we constructed a brief questionnaire (Q1-Q14). After the participants were informed that placebos were pills with no active medication inside of them and told that the assignment to each treatment was random and not related to their own preferences, they were asked to complete a brief questionnaire.

### Statistical Analysis

Descriptive analyses were performed to investigate the demographic and clinical variables. To compare the main demographic and clinical characteristics between groups, an unpaired Student's t-test was performed to analyze the continuous variables, and a chi-square test was performed to analyze the categorical variables. The data are presented in the form of the mean ± SD. After adjusting for confounding factors (age, gender, amount of education, duration of illness, CGI-S score, and GAF score), we conducted a multivariate logistic regression analysis with a forward selection method to assess the influence of attitude (Q2-Q14) toward placebo-controlled clinical trials as a predictor of participation in placebo-controlled clinical trials (Q1). A value of p < 0.05 was considered significant. The data were analyzed using the PASW Statistics PC software for Windows, Version 18.0.0 (SPSS Inc., Chicago, IL, USA).

## Results

The mean age of the full sample was 47.7±13.2 years old, the mean number of years of education was 11.4±2.0 years, and the mean duration of illness was 20.0±13.1 years. The average CGI-S and GAF scores were 4.0±0.8 and 57.9±14.3, respectively. Among the participants, 66.9% of the patients stated that they would not be willing to participate in a placebo-controlled clinical trial (Q1) (Table 1).

**Table 1:** Attitudes toward Participation in Placebo-Controlled Clinical Trials among Patients with Schizophrenia or Schizoaffective Disorder.

		Yes	No
Q1	Would you be willing to participate in a placebo-controlled clinical trial?	33.1%(83/251)	66.9%(168/251)
Q2	Do you think that it takes a lot of time (for medical examinations) to participate in a placebo-controlled clinical trial?	33.3%(84/252)	66.7%(168/252)
Q3	Do you want to try a new drug that could possibly improve your disease?	42.3%(107/253)	57.7%(146/253)
Q4	Do you think that placebo might worsen or slow improvement in your condition?	39.8%(100/251)	60.2%(151/251)
Q5	Do you think that your disease does not improve even when taking a new drug?	29.7%(74/249)	70.3%(175/249)
Q6	When you participate in a placebo-controlled clinical trial, do you think that you would receive more medical care?	43.8%(110/251)	56.2%(141/251)
Q7	When you participate in a placebo-controlled clinical trial, do you think that you would be able to talk to your physician more often?	43.4%(109/251)	56.6%(142/251)
Q8	When you participate in a placebo-controlled clinical trial, do you think that you would be guaranteed to be treated in this hospital?	48.2%(121/251)	51.8%(130/251)
Q9	Do you think that your doctor is pleased when you participate in a placebo-controlled clinical trial?	39.9%(99/248)	60.1%(149/248)
Q10	If your family or friends encouraged you, would you participate in a placebo-controlled clinical trial?	44.2%(111/251)	55.8%(140/251)
Q11	Have you ever participated in a clinical trial before?	8.0%(20/251)	92.0%(231/251)
Q12	In principle, do you approve of clinical trials?	61.8%(152/248)	38.2%(96/248)
Q13	Do you think that long-term medical treatment is necessary for you?	22.3%(56/251)	77.7%(195/251)
Q14	Do you want to support the development of new drugs?	29.3%(73/249)	70.7%(176/249)

When analyzing the influence of sociodemographic data (Table 2) on the willingness to participate in placebo-controlled clinical trials (Q1), we found that younger patients tended to be reluctant to participate in such trials (p = 0.056). No other differences in any other characteristics were observed.

**Table 2:** Characteristics according to decisions of whether to participate in placebo-controlled clinical trials.

	Patients' willingness to participate		p value
	Yes	No	
Age	50.0±13.3	46.6±13.0	0.056
Gender	Male 50, Female 33	Male 80, Female 88	0.239
Amount of education	11.5±1.9	11.4±2.1	0.945
Duration of illness	21.4±14.0	19.3±12.6	0.262
CGI-S	4.0±0.8	3.9±0.8	0.619
GAF	56.0±15.2	58.9±13.8	0.140

To assess the influence of attitudes (Q2-Q14) toward placebo-controlled clinical trials as a predictor of participation in placebo-controlled clinical trials (Q1), we performed a multivariate logistic regression analysis with a forward selection method. Responses to Q2 (belief of additional time required for medical examinations), Q3 (expectation of disease improvement), Q8 (guarantee of hospital treatment continuation), and Q10 (encouragement from family or friends) were significantly associated with the decision to participate in placebo-controlled clinical trials (Table 3).

**Table 3:** Factors associated with decisions of whether to participate in placebo-controlled clinical trials.

	B	Standard Error	Wald value	P value	Odds Ratio
Q2	-1.41	0.42	11.4	0.001	0.25 (0.11–0.55)
Q3	1.23	0.49	6.4	0.012	3.43 (1.32–8.96)
Q8	1.44	0.42	11.9	0.001	4.21 (1.86–9.53)
Q10	1.53	0.43	12.9	<0.001	4.59 (2.00–10.56)

After adjusting for confounding factors (age, gender, amount of education, duration of illness, CGI-S score, and GAF score), we performed a multivariate logistic regression analysis with a forward selection method to assess the influence of attitudes (Q2-Q14) toward placebo-controlled clinical trials as a predictor of participation in placebo-controlled clinical trials (Q1).

## Discussion

The present study of the attitudes of patients with schizophrenia toward placebo-controlled clinical trials is the first such survey of an Asian population. In our survey, although more than half of the patients (62%) stated that they approve of clinical trials in principle, only 33% of the respondents reported that they would be willing to participate in placebo-controlled clinical trials. An expectation of disease improvement (Q3), a guarantee of hospital treatment continuation (Q8), and encouragement from family or friends (Q10) were associated with a willingness to participate, whereas a belief of additional time required for medical examinations (Q2) was associated with non-participation. A previous study conducted in Austria [15] reported that 44% of the respondents were willing to participate in a placebo-controlled clinical trial. The reasons most often given by the patients who were willing to participate in such a study were (1) the wish to support the development of new drugs, (2) the possibility of remaining unmedicated, and (3) the desire to receive more medical care. In another study [17], the attitudes of patients with schizophrenia toward four hypothetical research protocols were assessed. The patients perceived significantly greater harm in medication washout or placebo treatment, and they were less willing to enroll in protocols perceived as more harmful. Furthermore, the patients indicated that doctor recommendations, monetary incentives, and, to a lesser extent, family preferences had a mild influence on their participation decisions. Schäfer and colleagues [18] evaluated the attitudes toward psychiatric research among patients with schizophrenia or depression in 7 European countries. They showed that most patients (98%) approved of psychiatric research and the reasons to participate were mainly altruistic. In the same study, expectation to receive a feedback of the studies' results was significantly more expressed by patients with schizophrenia as compared to depressive patients. With respect to schizophrenia researchers’ attitudes toward placebo-controlled clinical trials in Europe [19], willingness to participate in such clinical trials involving acutely ill patients was expressed by 30%, and 34% said that they would perform such a study in maintenance treatment. In response to the question of whether they believed that their local ethics committee would permit a placebo-controlled clinical trial, 24% answered yes for acute studies and 29% for maintenance studies.

Article 33 of the Declaration of Helsinki [16] states that "where no proven intervention exists, the use of placebo or no intervention is acceptable". Although placebo-controlled clinical trials are acceptable if there are good reasons for placebo use or if the condition being studied is "minor" and the additional risk is negligible, controversy remains. The finding that more than 50% of patients would not be willing to consent to a potential placebo-controlled study also raises doubts regarding the generalizability of data obtained by these studies [15]. Both the willingness of patients and the readiness of psychiatrists to include patients in such trials appear to result in selection bias [19]. Although we could not find a significant relationship between possible confounding factors (age, gender, amount of education, duration of illness, CGI-S score, and GAF score) and the willingness to participate in placebo-controlled clinical trials, the possibility of selection bias in placebo-controlled clinical trials could not be completely eliminated [20]. Furthermore, a higher dropout rate for placebo-controlled clinical trials [21] compared with active-control clinical trials could hamper the generalizability of data obtained by these studies.

Although active-control clinical trials have been suggested as an alternative method for licensing new drugs [22], it is difficult to prove that a new treatment is better than a currently available treatment in active-control clinical trials. Researchers prefer situations in which studies demonstrate that a new drug is equally effective as the active-control drug. However, some medications that are currently available and considered effective have not always been proven as such in comparison to placebo [22, 23]. Furthermore, the instruments used for measurement in psychiatry are often subject to measurement error due to the nature of observation, self-report or interview.

To date, several studies have been conducted to clarify whether placebo could cause additional risks of serious or irreversible harm. Some studies [22–24] have found no differences in the clinical course or social outcomes between patient-subjects randomly assigned to placebo or to continuous medication. However, Zipursky and colleagues [25] reported that one-year recurrence rate following discontinuation of antipsychotic medication was 77%. In addition, a recent study conducted in South Africa [26] found that placebo-treated patients who experience a relapse have slightly smaller improvements in the positive and negative syndrome scale (PANSS) total score upon reintroduction of paliperidone palmitate compared with the improvements observed upon initial introduction. Although the difference was small, treatment refractoriness may evolve in patients after relapse.

The current findings must be cautiously interpreted for the following reasons. First, the cross-sectional nature of the study does not allow for causal assumptions about attitude and the willingness to participate in placebo-controlled clinical trials. Second, some parameters that may contribute to the willingness to participate in placebo-controlled clinical trials were not included in this study, such as competence to consent, and socioeconomic status. Especially, the competence to consent may be an important factor. Future studies using a structured interview that assesses the competence to consent are needed. Third, symptom severity was assessed by CGI-S or GAF, rather than by PANSS or a clinician-administered structured diagnostic interview.

In conclusion, fewer than half of the respondents in this study stated that they would be willing to participate in placebo-controlled clinical trials. Interpreting the results of placebo-controlled clinical trials could thus be hampered by the potential for selection bias.
